# Summary

**Published:** 1882-03

**Authors:** 


### Summary.

RECENT CRIMES.—There would seem to be some reason to fear that the progress of civilization is travelling the course of a vicious circle. Side by side with tokens of intellectual advance and moral enlightenment are to be found unmistakable evidences of the rapid and marked development of exceptionally evil quality in the natures of individuals who have certainly not been left out of the swiftly moving current of progress, but have turned its advantages to their own and their neighbor's injury.

A girl thirteen years of age (Messenger), having more than average intelligence, drowned a child committed to her charge, apparently with no stronger motive than the desire to save herself the trouble of nursing it. A young man (Lefroy) in his twenty-second year, fairly educated, if not in a certain way gifted has elaborately planned and brutally executed the murder of an old man, solely, as it would appear, to possess himself of a small sum of money to supply his immediate necessities. And perhaps for the first time in modern history, a party of young girls barely in their teens, would seem to have organized themselves as semi-professional house-breakers.

These crimes shock the moral instinct of the community, and the manner of their commission, and the bearing of the criminals after detection, occasion astonishment. Let us see whether what we think strange is not, in fact, perfectly natural—that is to say, natural to the abnormal conditions. There have, of course, in all ages and stages of natural progress and social development, been isolated instances of precocious wickedness; but we think it must be conceded, on consideration, that the story of recent crime—

from which we have cited only three of the latest examples—furnishes proof of exceptional depravity.

In truth, without adducing the detailed arguments by which we arrive at this conclusion, it may be suggested that the general effect of our high-pressure method of forcing the growth of intelligence by education, must needs accelerate the development of bad as well as good mental organizations. The sun shines and the rain falls on the evil as well as the good, and the total result must be to produce a sprinkling of preternaturally developed offences and offenders.

The psychologist who studies mind in the light of physiology—and how or where else can it be studied ?—knows that it is from the evil tree we get evil fruit, and the stronger the fostering influence brought to bear on the population as a whole, the longer must be the total number of bad fruit-bearing growths which will be brought into prominent activity. Those who regard education as a panacea for all the faults of human nature, must be woefully ignorant of its power and method of working. If it leads out the good, it must also lead out the evil.—*The London Lancet*, February.

One Unquestionable Result of the Guiteau Trial.—At the commencement of the trial of Guiteau, there was an almost universal confidence in the popular, as well as in the professional mind, as to the judicial ability of the medical profession to diagnosticate, with reasonable certainty, the character and degrees of mental alienation presented for examination. Such has been the record and the law; such the conviction and endorsement of the medical profession; such the custom and the rule with bench and bar; while the clergy have taken such rulings as absolute, and the people have said it must be so, if the doctors have said so.

What is the conviction of the profession and of the people today, after giving the so-termed medical experts a fair hearing, and their testimony an honest consideration ? It is that the claimed ability of the profession to determine these questions is absolutely untenable; that their testimonies, when put to the test, have been so different to the evidence expected and assumed, as to make their claim an absurdity, and to convert their proceedings into an absolute farce.

Some of the "experts" regard insanity as a disease of the mind, the brain being sound. Some assert that insanity is a disease of the brain, the mind being only functionally disturbed. Some that insanity is not a disease either of the mind or the brain, but that it comes from without, and not from within; that it is eccentric and not centric in origin. Some believe in moral insanity. Some declare this claim to be an absurdity.

But this forensic drama as a whole, has been lamentable, pitiable and mortifying. It has made universal skepticism in regard to expert testimony in psychiatry an unquestionable result of the Guiteau trial.—*Gaillard's Medical Journal*, January.

What seems so anomalous in the testimony of the experts looks perfectly natural upon further inquiry. The study of mind, psychology or psychiatry, has not yet had time to become a science. The first work on "Comparative Psychology: Mind in the Lower Animals," by W. Lander Linsay, was published in this country two years ago, and the valuable Works of Henry Maudsley are nearly as recent. Yet, if we wish to study the biological laws which have presided over the evolution of mind in man, no work has ever been written on this subject; nothing but some suggestions made by Herbert Spencer, which assume a more important form in the speculations of Edward B. Tylor in his "Anthropology" just published. So that the assertions of most so-called experts are based on their observation of certain facts, too scanty to give rise to useful generalization, and the more worthless because the observers have no principles to be guided by. It is about time that the diseases of the mind should be studied by the profession, and treated at home if possible.

THE PROGRESS OF THERAPEUTICS.—It seems to be in the nature of scientific study to run for a time in certain special lines, to the neglect of others—first one branch and then another being pushed far out in advance of the general progress of the science, and receiving, for the time, almost the undivided attention of investigation. This has been pre-eminently the case in medicine. Anatomy, physiology, physical diagnosis, and lastly pathology, have each in their turn almost monopolized the study of those who gave direction to medical thought.

It is only within a comparatively recent period that any systematic effort has been made to do for therapeutics what so many and such zealous workers have been doing for the other departments of medical science.

Medicines are beginning to be classified according to their primary action rather than their secondary results, and the old loose and general designation, such as stimulants, tonics, antispasmodics, evacuants, etc., are giving way to terms expressing the action upon special centres, such as cardiac, vaso-motor, cerebral, spinal, cerebro-spinal, excitants and depressants, etc. We now ask ourselves, not what is good for the disease in hand? but, what are the morbid conditions which find expression in the disease? How far are they capable of being modified by drugs? and lastly, what medicines will be best adapted to effect these modifications? This series of questions shows not only the dependance of rational therapeutics upon pathology, but also that pathology cannot be brought to the aid of therapeutics except through a knowledge of the initial action of drugs upon the several parts of the body. Thus, if our knowledge of pathology enables to determine that there is spasm of the vessels in a given part, and experimentation has taught us that a certain medicine will relax the vessels of that part, we have an exact indication for the use of that medicine such as no amount of merely clinical experience could afford us.

In a general way it has, of course, always been known that emetics act upon the stomach, and cathartics upon the bowels; that the skin is stimulated by diaphoretics, and the urine increased by diuretics; and the use of drugs with the purpose of "stirring up the liver" is as old as medicine itself. But in later times it has been found that this principle of elective action has a much wider range than was supposed, and is much more definite and exact in its application. Thus, the action of ergot upon unstriped muscular fiber makes it much more than a "parturient," and that of aconite, upon the vaso-motor centers makes it more than a simple diaphoretic or febrifuge. The specific action of the solanaceæ upon the third nerve, and of aconitia upon the fifth, of quebracho upon the respiratory centers, and of digitalis upon the cardiac, are instances in point. Nay, sometimes special branches of a nerve are affected to the exclusion of other branches of the same nerve, as, for example, the twig to the levator palpebræ, which is paralyzed by gelseminum.

The Cartwright lectures by Professor Bartholow, along with much that is original, give an admirable summing up of what has been accomplished of late in this most interesting field (the antagonism of medicines), while Fothergill's little treatise on the same subject is full of valuable information.

Going back only so short a period as twenty years, we have had within that time many new and most valuable medicines added to our list. Among these may be mentioned the bromides, iodoform, chloral, carbolic, salicylic and boracic acids and their compounds, thymol, amyl nitrite, guarana, jaborandi, quebracho, etc. Hypodermic medication with its multiform uses, and rectal alimentation, have been added to our resources within this period.

The use of small and frequently repeated doses, which has been introduced within a few years, seems to have much to recommend t, especially in the case of drugs which are rapidly eliminated. If the action of a medicine is needed at all, it appears more reasonable that it should be sustained at a nearly even intensity, rather than that the field should be occupied alternately by the remedy and the disease.

On the whole, whether we consider the many valuable additions to our list of remedies, or the more exact knowledge which has been obtained of the action of medicines, the outlook for therapeutics is full of promise.—*Medical Record*, Feb. 11.

